# Supplementary material for: Biological Control Activity of Plant Growth Promoting Rhizobacteria Burkholderia contaminans AY001 against Tomato Fusarium Wilt and Bacterial Speck Diseases
Source: Biology (Basel). 2022 Apr 18;11(4):619. doi: 10.3390/biology11040619 (PMC9028202; doi:10.3390/biology11040619)
Supplement: Supplementary file 1 [file biology-11-00619-s001.zip › biology-1673890-supplementary.pdf]

Supplementary Table S1. The list of Real-time qRT-PCR primers used in this study

Gene	Primer Sequence (5’ to 3’)		Amplicon size (bp)	Refrence
Actin	F:<br>R:	TCAGCAACTGGGATGATATG<br>TTAGGGTTGAGAGGTGCTTC	112	Milling et al., 2011.
PIN2	F:<br>R:	CATCTTCTGGATTGCCCA<br>ACACACAACTTGATGCCCAC	106	Li et al., 2014.
LapA	F:<br>R:	GGGACTAATGATGTTTGGAA<br>GTGGCAATTTTATTTAGGCA	109	Li et al., 2014.
ACO1	F:<br>R:	TTGCTCATTTCCTTTGTGGA<br>GGAAGCTAGCAAAGCAAACC	122	Jia et al., 2013.

Supplementary Table S2. Summary of PGPR-related trait of AY1001

PGP trait	AY001
Phosphate solubilization	+
Protease activity	+
Amylase activity	-
Siderophore	+
Nitrogen fixation	+
Zinc solubilization	+
Cellulase activity	-
Chitinase activity	-
IAA production	+
Ammonia production	+

Supplementary Table S3. Qualitative analysis of zinc and phosphate solubilization and protease efficiency of AY001 at 10 dai (Mean ± SD)

Incubation time	Total diameter (mm) (Colony + Halo zone)			Solubilization index (SI) and Hydrolysis index (HI)		
	Zinc solubilization	Phosphate solubilization	Protease activity	Zinc solubilization	Phosphate solubilization	Protease activity
Day 3	19.85±0.09	12.67±1.15	15.83±0.76	1.99	1.27	1.58
Day 5	22±0.09	16.67±1.26	33.83±0.29	2.13	1.52	2.78
Day 7	24.96±0.03	19.33±2.25	39.5±0.5	2.24	1.73	2.96
Day 10	26.5±0.13	24.83±1.04	46.83±0.29	2.24	2.22	3.01

References

Milling, A., Babujee, L., and Allen, C. (2011). *Ralstonia solanacearum* Extracellular Polysaccharide Is a Specific Elicitor of Defense Responses in Wilt-Resistant Tomato Plants. *PLoS One* 6, e15853. doi: 10.1371/journal.pone.0015853

Li, X., Huang, L., Zhang, Y. et al. (2014). Tomato SR/CAMTA transcription factors SlSR1 and SlSR3L negatively regulate disease resistance response and SlSR1L positively modulates drought stress tolerance. *BMC Plant Biol*. 14, 286. doi: 10.1186/s12870-014-0286-3

Jia, C., Zhang, L., Liu, L., Wang, J., Li, C., and Wang, Q. (2013). Multiple phytohormone signalling pathways modulate susceptibility of tomato plants to *Alternaria alternata* f. sp. *lycopersici*. *J. Exp. Bot*. 64, 637–650, doi: 10.1093/jxb/ers360

A

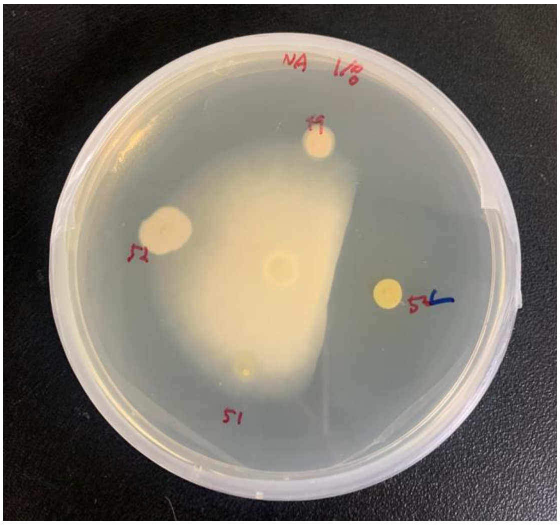

B

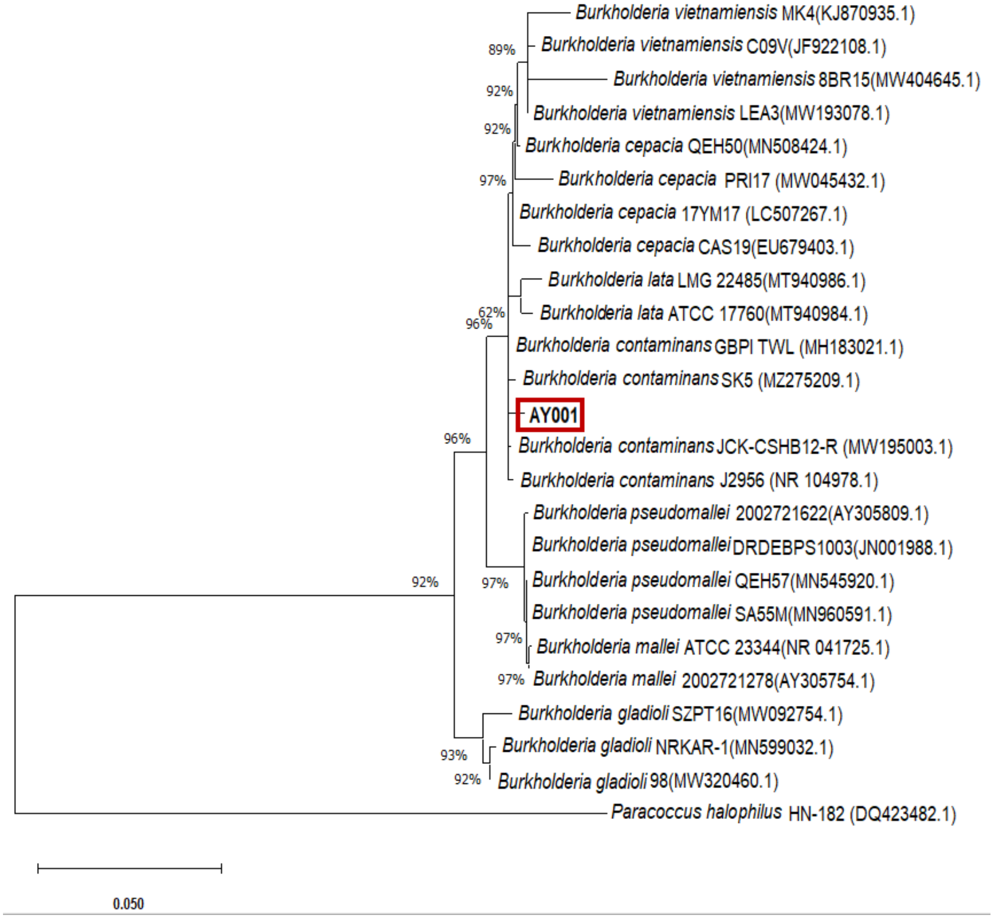

Supplementary Figure S1. Isolation and identification of antagonistic bacterium AY001 against *Fusarium oxysporum* f.sp. *lycopersici* (*FOL*). **(A)** Isolation of AY001 showing antagonistic effect against FOL from PDA media. **(B)** Molecular identification of AY001 as *Burkholderia contaminans*. Phylogenetic analysis of 16s rDNA of AY001 with other *Burkholderia* spp. Phylogenetic tree were generated by Maximum Likelihood method analysis of 16S rRNA gene nucleotide sequences of *Burkholderia* species in MEGA X program.
